# Blood speckle imaging compared with conventional Doppler ultrasound for transvalvular pressure drop estimation in an aortic flow phantom

**DOI:** 10.1186/s12947-022-00286-1

**Published:** 2022-07-16

**Authors:** Cameron Dockerill, Harminder Gill, Joao Filipe Fernandes, Amanda Q. X. Nio, Ronak Rajani, Pablo Lamata

**Affiliations:** 1 School of Biomedical Engineering & Imaging Sciences, King’s College London, London, UK; 2 Cardiology Department, Guy’s and St Thomas’ NHS Foundation Trust, London, UK

**Keywords:** Blood speckle imaging, Doppler, Ultrasound, Echocardiography, Transvalvular pressure drop, Aortic stenosis

## Abstract

**Background:**

Transvalvular pressure drops are assessed using Doppler echocardiography for the diagnosis of heart valve disease. However, this method is highly user-dependent and may overestimate transvalvular pressure drops by up to 54%. This work aimed to assess transvalvular pressure drops using velocity fields derived from blood speckle imaging (BSI), as a potential alternative to Doppler.

**Methods:**

A silicone 3D-printed aortic valve model, segmented from a healthy CT scan, was placed within a silicone tube. A CardioFlow 5000MR flow pump was used to circulate blood mimicking fluid to create eight different stenotic conditions. Eight PendoTech pressure sensors were embedded along the tube wall to record ground-truth pressures (10 kHz). The simplified Bernoulli equation with measured probe angle correction was used to estimate pressure drop from maximum velocity values acquired across the valve using Doppler and BSI with a GE Vivid E95 ultrasound machine and 6S-D cardiac phased array transducer.

**Results:**

There were no significant differences between pressure drops estimated by Doppler, BSI and ground-truth at the lowest stenotic condition (10.4 ± 1.76, 10.3 ± 1.63 vs. 10.5 ± 1.00 mmHg, respectively; *p* > 0.05). Significant differences were observed between the pressure drops estimated by the three methods at the greatest stenotic condition (26.4 ± 1.52, 14.5 ± 2.14 vs. 20.9 ± 1.92 mmHg for Doppler, BSI and ground-truth, respectively; *p* < 0.05). Across all conditions, Doppler overestimated pressure drop (Bias = 3.92 mmHg), while BSI underestimated pressure drop (Bias = -3.31 mmHg).

**Conclusions:**

BSI accurately estimated pressure drops only up to 10.5 mmHg in controlled phantom conditions of low stenotic burden. Doppler overestimated pressure drops of 20.9 mmHg. Although BSI offers a number of theoretical advantages to conventional Doppler echocardiography, further refinements and clinical studies are required with BSI before it can be used to improve transvalvular pressure drop estimation in the clinical evaluation of aortic stenosis.

**Supplementary Information:**

The online version contains supplementary material available at 10.1186/s12947-022-00286-1.

## Background

Doppler echocardiography is routinely used in clinical practice to assess the severity of aortic stenosis. The maximum velocity of blood flow through the aortic valve during systole is recorded, and the simplified Bernoulli equation is used to estimate the transvalvular pressure drop (a more accurate term to the widely used *gradient*) across the valve [1]. This technique is preferred to cardiac catheterisation as it is non-invasive, widely accessible and inexpensive [2].

Despite this, applying the simplified Bernoulli equation, as is the case in Doppler echocardiography, has been shown to overestimate transvalvular pressure drops by up to 54% when compared to the equation accounting for the complete haemodynamic profile at the point of maximum constriction [3]: taking peak velocity events in the Bernoulli formulation ignores the momentum of blood flow across the entire vascular cross-section that is key to estimate the actual pressure drop. In addition, pressure drop estimation using Doppler echocardiography is highly user-dependent. If the angle of insonation is not fully aligned with the direction of blood flow, the maximum velocity will be missed [4]. Several non-invasive alternatives have been studied but are not yet applied clinically [5].

Blood speckle imaging (BSI) has recently emerged as an alternative methodology for the assessment of aortic stenosis severity [6, 7]. By the direct measurement and visualisation of blood vector velocity fields, captured at ultra-high frame rates in the kilohertz range [6, 8, 9], it has the potential to overcome the angle-dependence and acquisition of single peak velocities, which currently limit conventional Doppler echocardiography [3]. BSI utilises existing technology from tissue speckle-tracking that is commonly used to evaluate myocardial deformation [10]. A small image kernel is defined in the first image of the vessel and the same speckle signature is tracked in the following frame using a “best match” search algorithm. This is then repeated for a grid of measurements to quantify the velocity and direction of the blood flow [6, 11]. Acquiring blood flow velocity data in this way is advantageous as it potentially allows pressure drop to be calculated from velocity data across a cross-sectional profile [3], rather than from a single streamline in conventional Doppler echocardiography, although thisis not investigated in this report.

The principal aim of the current manuscript was to evaluate blood speckle imaging (BSI) and Doppler echocardiography for pressure drop estimations derived from maximum velocity values against ground-truth pressure sensors in a bespoke aortic phantom with a 3D-printed aortic valve at various flow rates.

## Materials and methods

### Pressure drop phantom

In order to investigate the accuracy and utility of novel techniques for pressure drop estimation, a bespoke aortic phantom was developed [12, 13]. The phantom was designed to simulate the human aorta, able to deform with pulsatile and constant flow conditions, allowing for comparison of novel pressure drop estimation techniques to ground-truth pressure drop data from pressure sensors across a 3D-printed aortic valve. Pressure drop values measured across valves manufactured using these methods, placed into the same phantom system were representative of those reported in vivo [13].

**Fig. 1:**
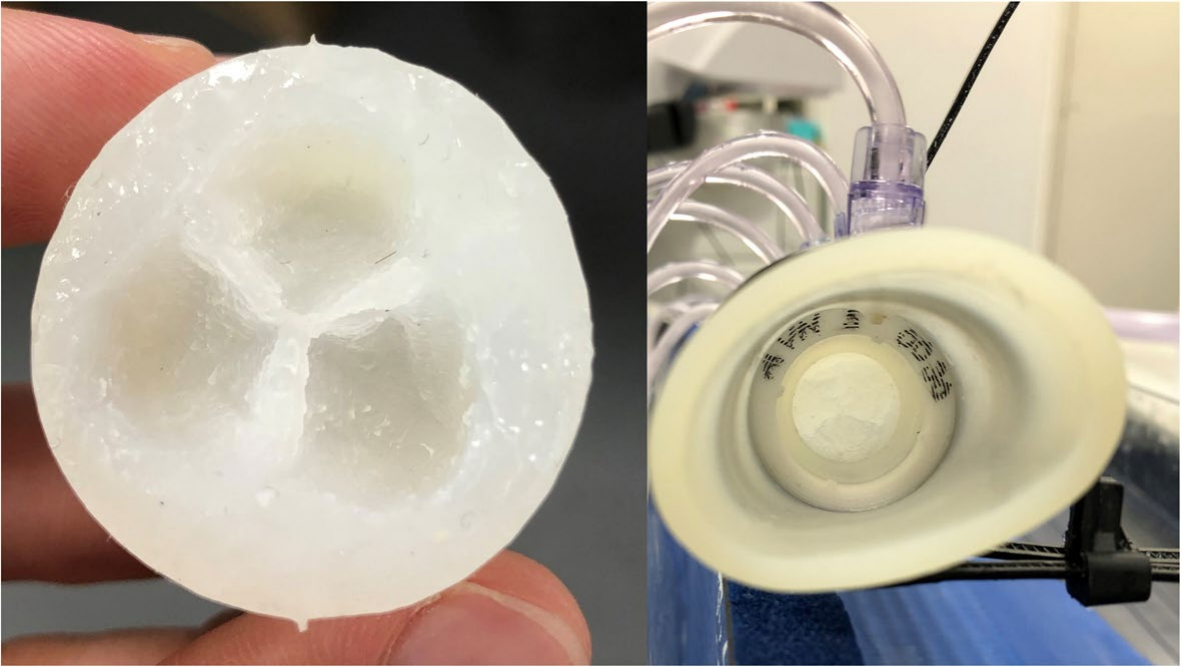
The Ecoflex 0030 silicone aortic valve used for the experiments. Pictured from the front, outside of tube (left) and from the back, in situ (right)

A silicone 3D-printed aortic valve model was placed within a semi-compliant silicone tube of 32 mm internal diameter, suspended in an acrylic box (Fig. 1). The valve was designed to resemble a healthy aortic valve, segmented from patient CT scans. The valve design was converted to a mould, before degassed Ecoflex 0030 silicone (Smooth-On Inc., Macungie, PE, USA) was poured in and left to cure. Valve mounts were also 3D printed using poly-lactic acid.

**Fig. 2:**
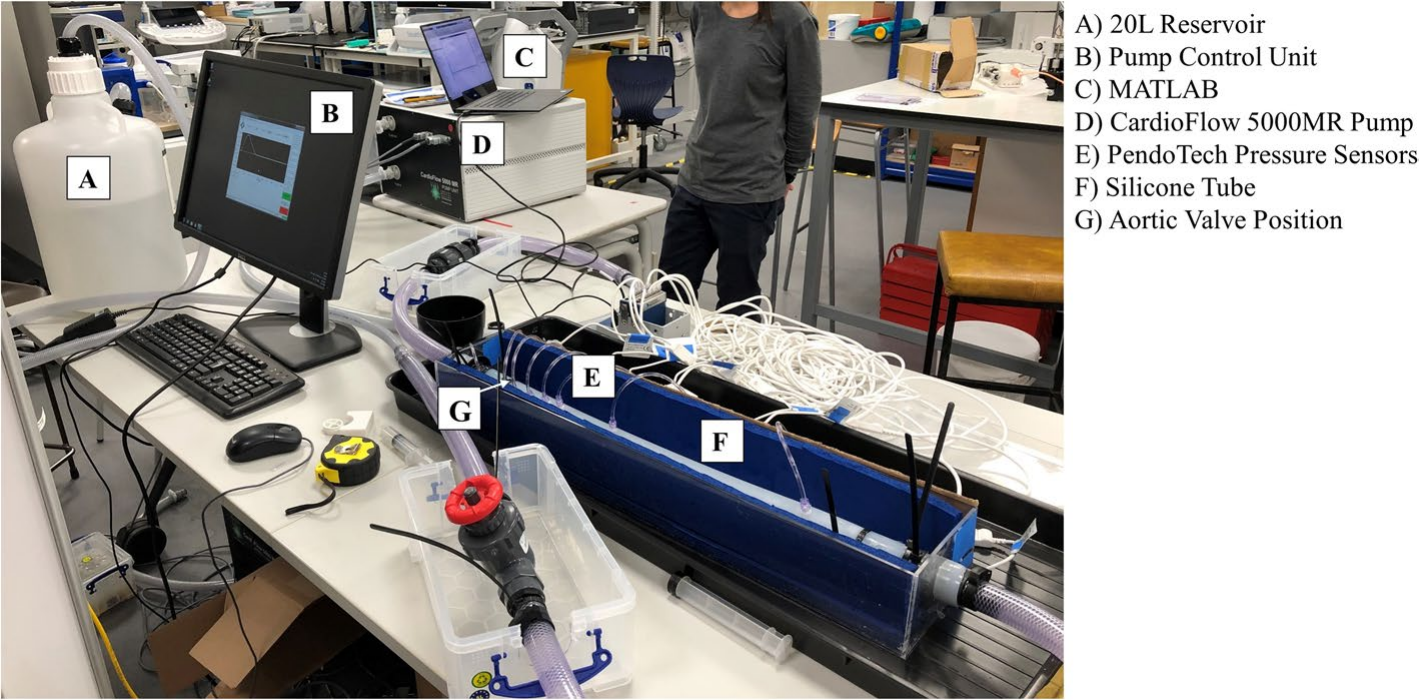
The phantom used for the velocity-based pressure drop estimation experiments

Eight PendoTech pressure sensors (PRESS-S-000 sensor, PendoTech, Princeton, NJ, USA) were embedded along the tube wall, 1 situated before the valve and 7 downstream. The position of each pressure sensor was decided aiming to both capture the event of maximum constriction (i.e. location of the vena contracta) and the distal net pressure drop (i.e. characterisation of the pressure recovery). The positions of the sensors, relative to the valve, were -3.0 cm, 1.5 cm, 3.0 cm, 5.0 cm, 7.5 cm, 10.0 cm, 20.0 cm and 50.0 cm. The sensors were calibrated and validated using a pressure catheter (Mikro-Cath, Millar Inc, Houston, TX, USA). The pressure sensors were wired to input modules of a data acquisition USB chassis (National Instruments, Austin, TX, USA) to record ground-truth pressures for the 8 locations along the silicone tube at a sampling frequency of 10 kHz (Fig. 2).

A 20 L external reservoir containing blood mimicking fluid [14] was connected to a CardioFlow 5000MR flow pump (Shelley Medical Imaging Technologies, Ontario, Canada). The pump was programmed, via control unit, to circulate approximately 15 L of blood mimicking fluid at eight different pump flow rates (100, 150, 200 and 250 mL/s, constant and pulsatile flows). Flow was maintained at a constant rate throughout each acquisition in the constant flow conditions. The pulsatile flow conditions were programmed to closely resemble the flow waveforms produced by the human heart, with fluctuations in pressure corresponding to systole and diastole.

### Velocity and ground truth pressure data acquisition and analysis

**Fig. 3:**
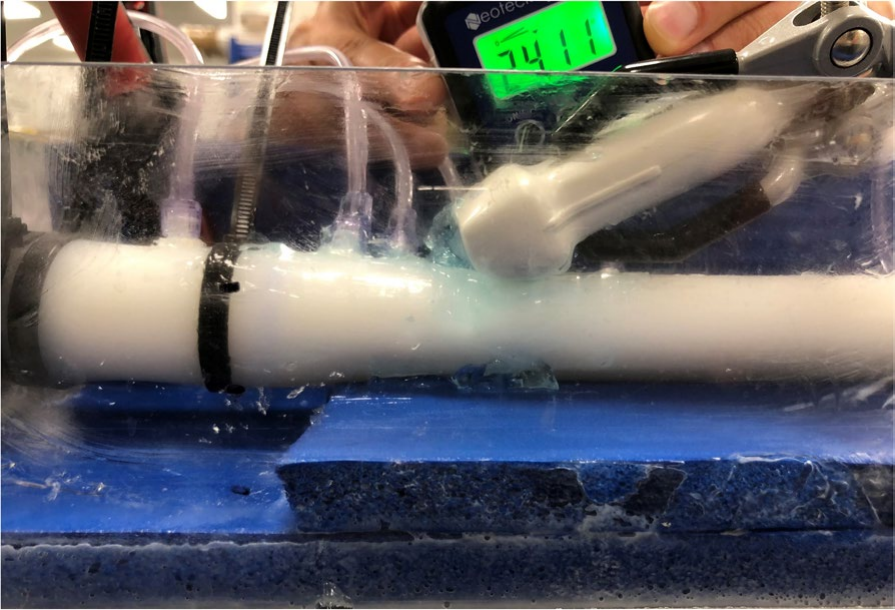
A photograph taken during the experiments, illustrating probe orientation and tilt angle measurement

Continuous wave Doppler and BSI data were acquired using a Vivid E95 ultrasound machine and 6S-D cardiac phased array transducer (GE Healthcare, Oslo, Norway) across the valve for each flow condition. Ground-truth pressure drop data were calculated using the methods outlined below. A metal clamp held the probe in a fixed position during all acquisitions. The tilt angle between the tube and probe was recorded and used for angle correction calculations (Fig. 3). Each experimental condition was repeated on a second day of experiments.

**Fig. 4:**
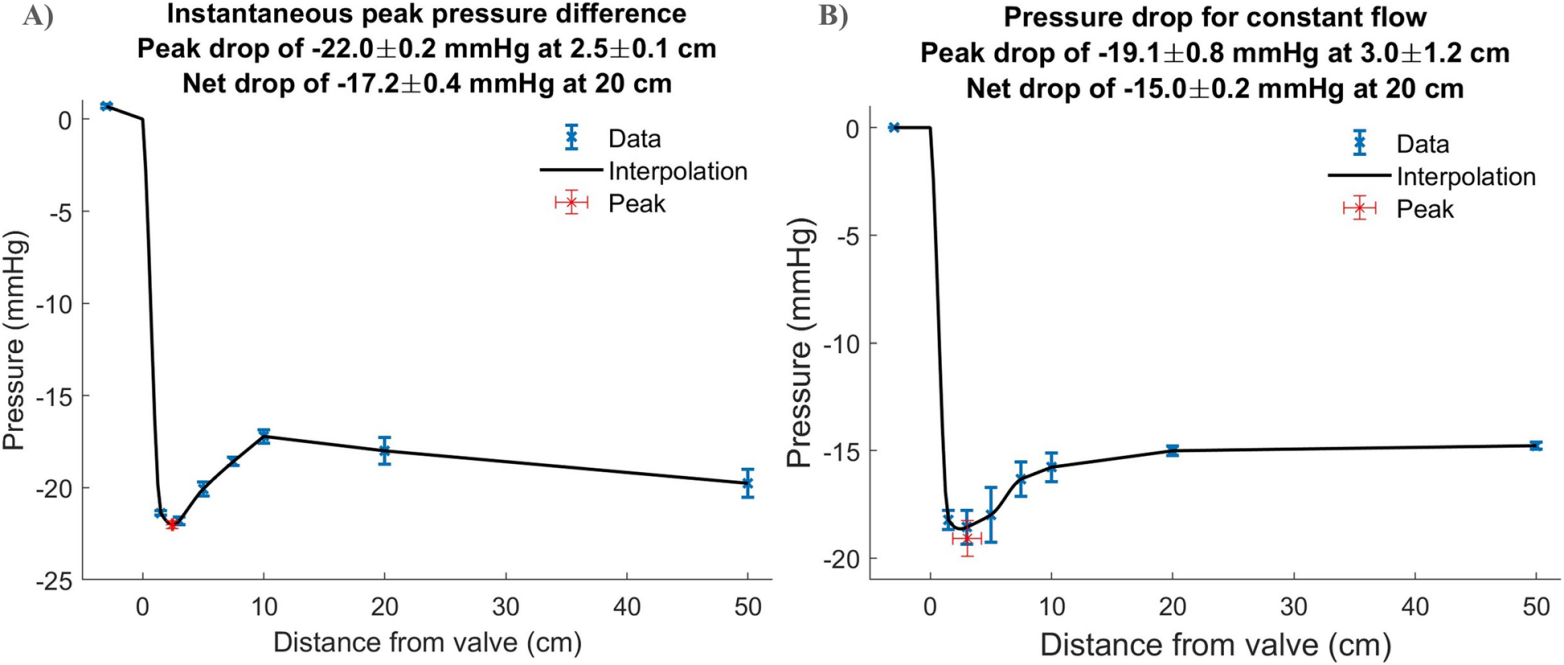
Example plots of the pressure drop measured by the pressure sensors at 250 mL/s **A** pulsatile flow **B** constant flow rates. Blue points and error bars represent mean ± standard deviation from the 8 pressure sensors. The red point represents the maximum pressure drop and its location, estimated using a modified Akima piecewise cubic Hermite interpolation between the valve and sensor 4

Pressure sensor data were extracted and analysed offline using MATLAB (Mathworks, Natick, MA, USA). To calibrate the pressure sensors for random error, the mean pressure across the 8 pressure sensors was calculated with static fluid in the phantom, before and after each experimental condition. For each condition, a correction was applied to each pressure sensor based on its deviation from this set mean. Pressure data in pulsatile conditions was enhanced with a Butterworth filter that reduced the random peaks and noise on the raw temporal transients of pressure data from each sensor. For each experimental condition and flow rate, pressure data were recorded over 8 s. The maximum number of available cycles were then segmented before the mean and standard deviation of the pressure transient were calculated. In most cases, 6 full cycles were used for these analyses. The instant of peak pressure difference between the valve and channels 2 or 3 was selected for further analysis. It was assumed that the pressure at valve level (0 cm) was the same as in Channel 1 (-3 cm) after correcting for a time shift to account for the time taken for the pulse wave to travel between Channel 1 and the valve. The mean and standard deviation values from each pressure sensor were then plotted against their physical position in the phantom, relative to the valve at point 0 cm. Modified Akima piecewise cubic Hermite interpolation was performed between the valve and channel 4, the region of the vena contracta, to estimate the maximum pressure drop and its location, relative to the valve (Fig. 4A). In constant flow conditions, a Butterworth filter was applied to the pressure signals before the mean and standard deviation values from each pressure sensor were then plotted against their physical position in the phantom. (Fig. 4B).

**Fig. 5:**
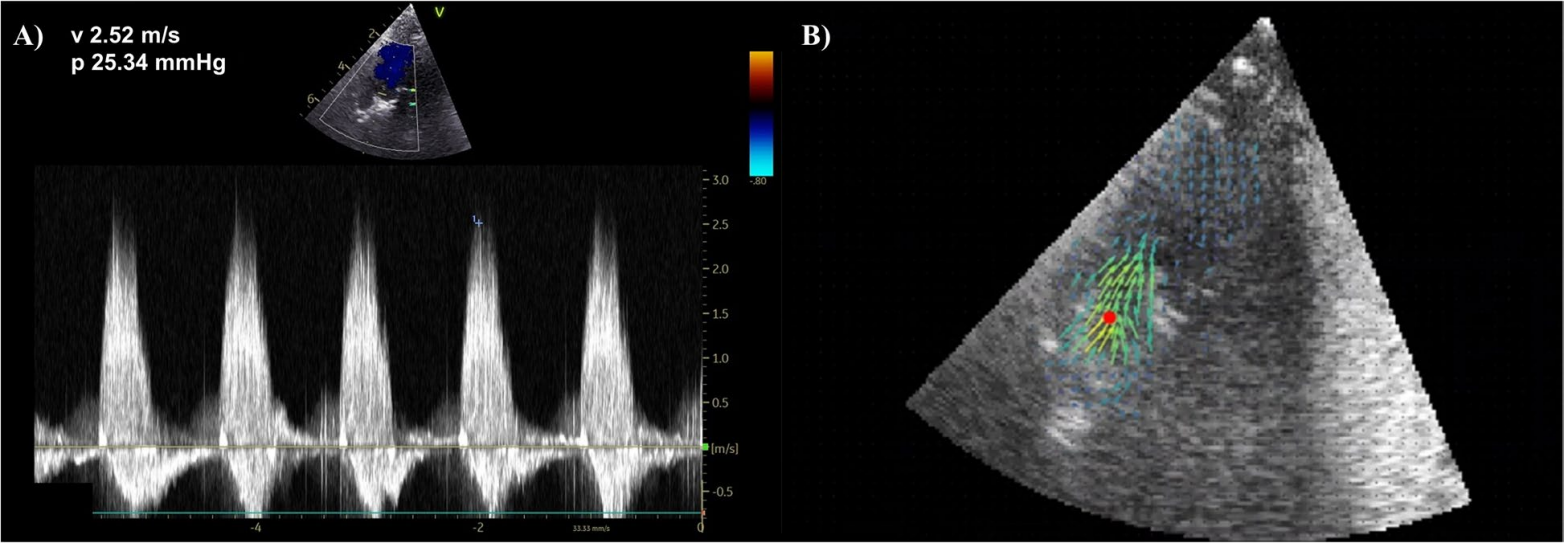
**A** Example continuous wave Doppler acquisition at 250 mL/s flow rate with manual measurement shown. **B** Example BSI velocity vector interface, showing the velocity of flow through the region of interest

To estimate maximum velocity using Doppler, a continuous wave acquisition was acquired with the cursor placed at the valve opening. Once acquired, the E95 machine was used to manually select the maximum velocity observed in the acquisition (Fig. 5A). To estimate maximum velocity using BSI, the blood speckle imaging setting was used on the Vivid E95 machine to acquire BSI velocity data at frame rates in the kilohertz range [6]. A movie containing examples of BSI acquisitions at the different flow rates can be viewed in the additional files (Additional File 1). The computation of the velocities acquired using BSI was performed with the manufacturer code for BSI velocity quantification (GE Healthcare, Oslo, Norway). The detection of peak velocities at the region of interest, positioned at the vena contracta, where velocity was maximal, was programmed in-house. Secondary validation was performed by an observer, using the interface shown in Fig. 5B to confirm that the maximum velocity vector was indeed observed within the expected region of interest where the jet after the valve is observed.

Pressure drop values were estimated from Doppler and BSI acquisitions by applying an angle correction, using the measured probe angle (Fig. 3), to the maximum velocity (m/s) value measured by each technique. Following this, the simplified Bernoulli formulation was applied to the angle-corrected velocity value to convert to transvalvular pressure drop (mmHg).

### Data and statistical analysis

Pressure drop and flow velocity data are presented as the mean ± standard deviation. To calculate the significance level of the values estimated by each technique, a paired two-tailed distribution t-test was used with a significance level of *p* < 0.05. To calculate the significance level of the bias in the Bland–Altman analyses, a one-sample two-tailed t-test was used with a significance level of *p* < 0.05 (Microsoft Excel, Microsoft Office Version 2102, Microsoft Corporation, Redmond, Washington, USA).

## Results

The probe angles were 24° and 40° on two days of experiments, respectively. The mean pressure drops across the valve, recorded by the ground-truth pressure sensors, were 9.65 ± 0.07, 12.3 ± 0.00, 16.2 ± 0.28 and 19.3 ± 0.21 mmHg under constant flow conditions and 11.3 ± 0.56, 14.3 ± 0.57, 17.9 ± 0.35 and 22.5 ± 0.71 mmHg under pulsatile conditions, at the flow rates investigated (100, 150, 200, 250 mL/s, respectively). There was no significant difference between the mean pressure drop values acquired under constant vs*.* pulsatile conditions (*p* > 0.05).

**Fig. 6:**
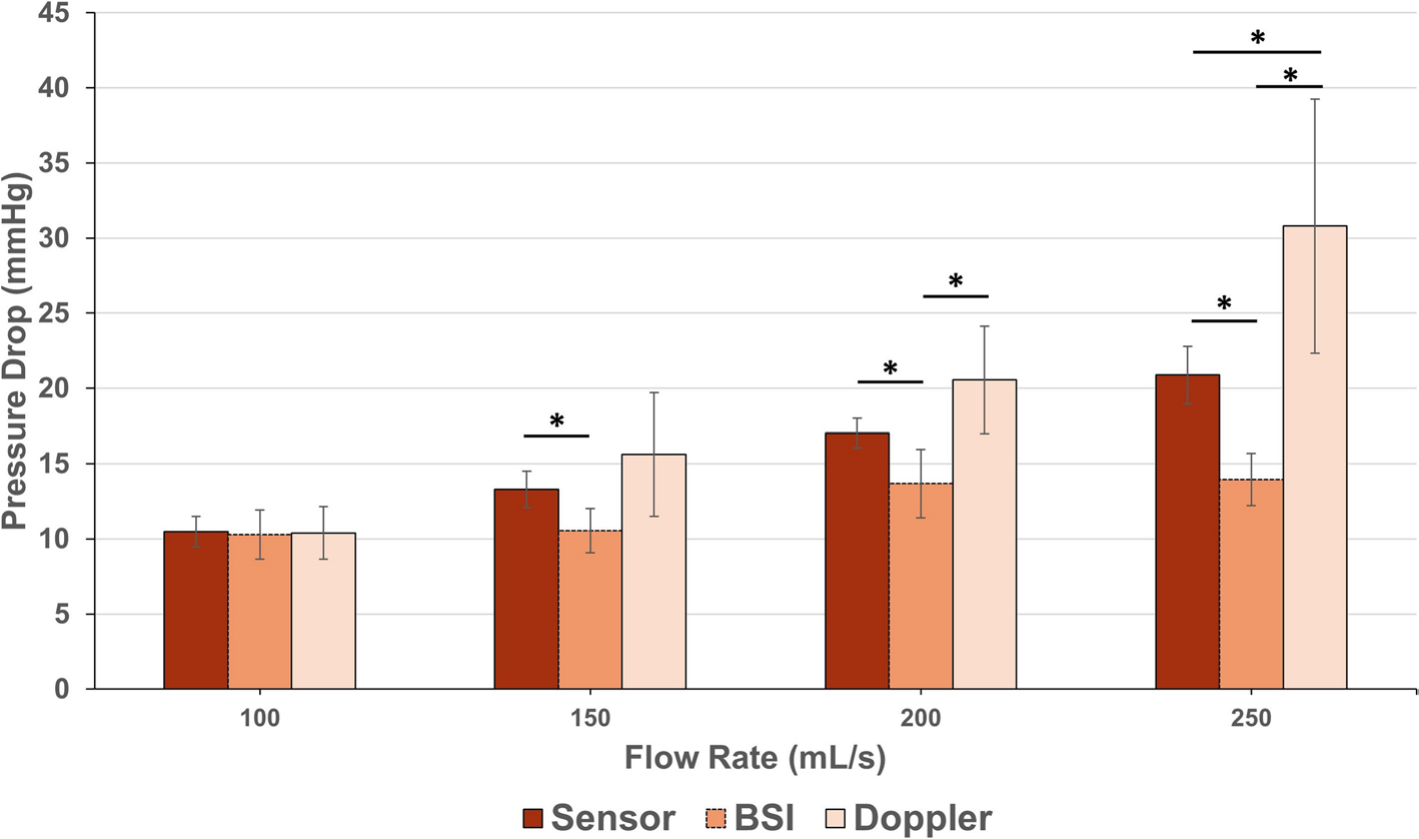
Angle-corrected pressure drop (simplified Bernoulli equation; mmHg) from BSI (orange dotted bars) and Doppler (pale bars), compared to ground truth acquired by sensors (dark bars). Data are presented as the mean estimated drop under each flow condition, grouping constant and pulsatile flows. Error bars represent ± standard deviation.* denotes statistical significance (*p* < 0.05)

The pressure drop values estimated across the 4 flow rates by the ground-truth pressure sensors, BSI and Doppler methods are presented in Fig. 6. No significant differences were observed between pressure drops estimated by Doppler, BSI and ground-truth sensors at the 100 mL/s pump flow rate (10.4 ± 1.76, 10.3 ± 1.63 vs. 10.5 ± 1.00 mmHg, respectively; *p* > 0.05).

Under the 150, 200 and 250 mL/s pump flow rates, pressure drops estimated by BSI (10.5 ± 1.47, 13.7 ± 2.27 and 14.5 ± 2.14 mmHg, respectively) were significantly lower than ground-truth pressure drops (13.3 ± 1.20, 17.0 ± 0.99 and 20.9 ± 1.92 mmHg, respectively; *p* < 0.05). On the other hand, pressure drops estimated from Doppler velocity data were significantly higher than ground-truth pressure drop under 250 mL/s pump flow conditions (26.4 ± 1.52 vs. 20.1 ± 1.78 mmHg, respectively; *p* < 0.05).

**Fig. 7:**
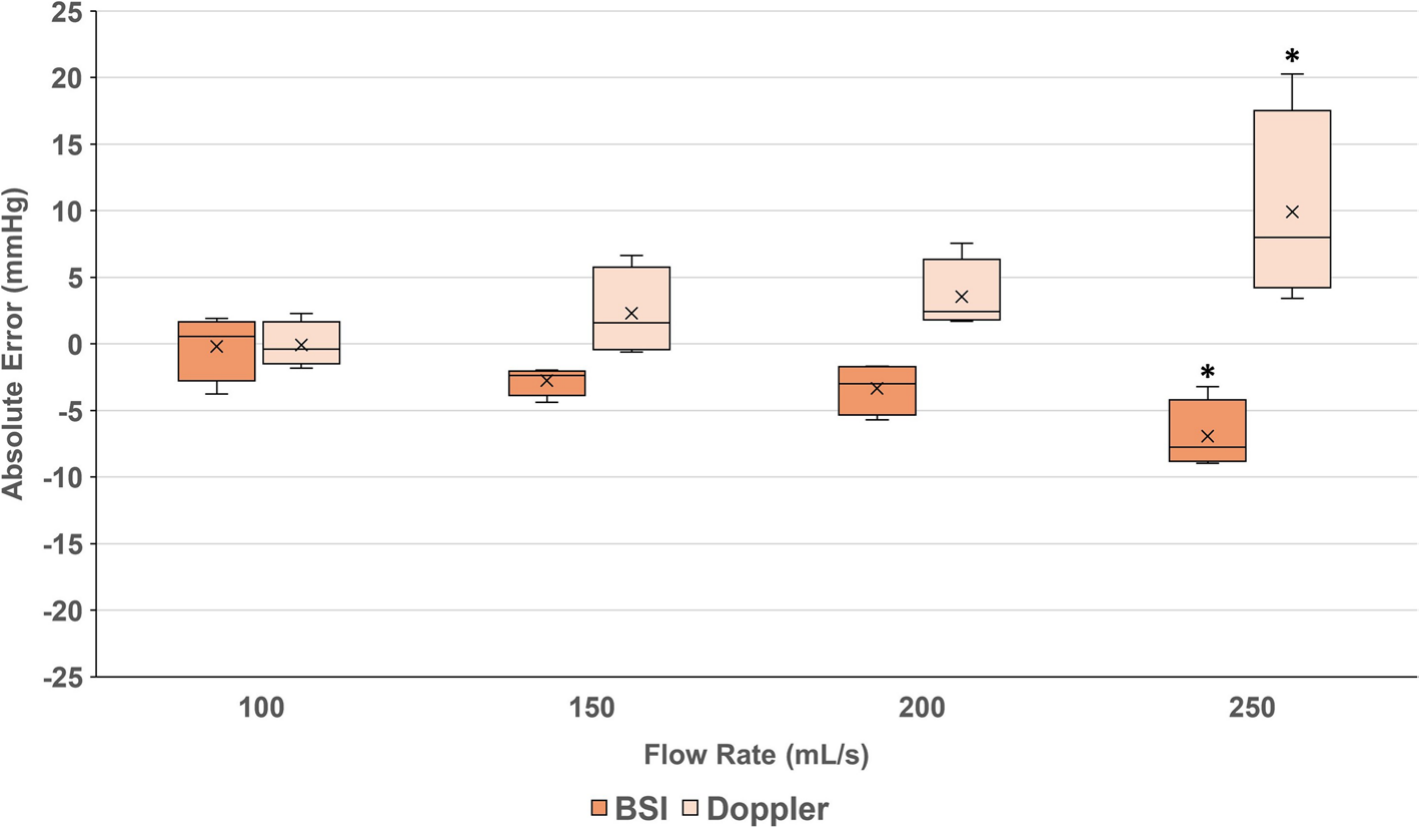
Absolute error of pressure drop estimations (mmHg) from BSI (orange bars) and Doppler (pale bars), compared to ground truth acquired by sensors. Data are presented as the mean value (cross), the median value (line), the upper and lower quartiles (box range) and the minimum and maximum values (whiskers) under each flow condition, grouping constant and pulsatile flows. * denotes statistical significance compared to the 100 mL/s flow rate ( *p* < 0.05)

The absolute errors in pressure drop estimation for each flow rate investigated, compared to the ground-truth pressure sensors, were –0.08 ± 1.72, 2.30 ± 3.30, 3.52 ± 2.72 and 9.91 ± 7.31 mmHg by Doppler and –0.19 ± 2.50, -2.76 ± 1.09, –3.42 ± 1.98 and –6.93 ± 2.58 mmHg by BSI at the flow rates investigated (100, 150, 200, 250 mL/s, respectively; Fig. 7). The absolute errors in pressure drop estimation by Doppler and BSI at the 250 mL/s flow rate were significantly higher and lower, respectively, than the absolute errors at the 100 mL/s flow rate (*p* < 0.05).

**Fig. 8:**
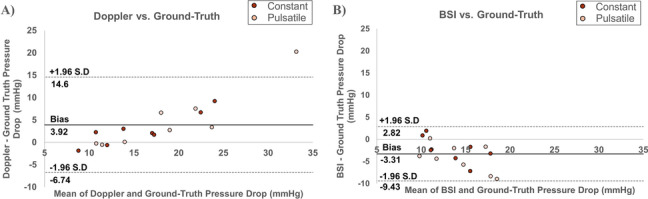
Bland–Altman plots illustrating the agreement between **A** Doppler vs. ground-truth and **B** BSI vs. ground-truth pressure drop estimations (*n* = 16). The solid line represents the bias between the 2 methods, while the 95% limits of agreement (± 1.96 standard deviation) are represented by the dashed lines

Bland–Altman analysis of the Doppler and BSI techniques, compared to ground-truth pressure drop are presented in Fig. 8 [15]. Figure 8A shows a statistically significant bias of pressure drop estimations made using Doppler, when compared to ground-truth pressure drop of 3.91 mmHg (*p* < 0.05). The upper limit of agreement was 14.6 mmHg and the lower limit was -6.74 mmHg (Fig. 8A). Figure 8B shows a statistically significant bias of pressure drop estimations made using BSI, when compared to ground-truth pressure drop of -3.31 mmHg (*p* < 0.05). The upper limit of agreement was 2.82 mmHg and the lower limit was -9.43 mmHg (Fig. 8B).

**Fig. 9:**
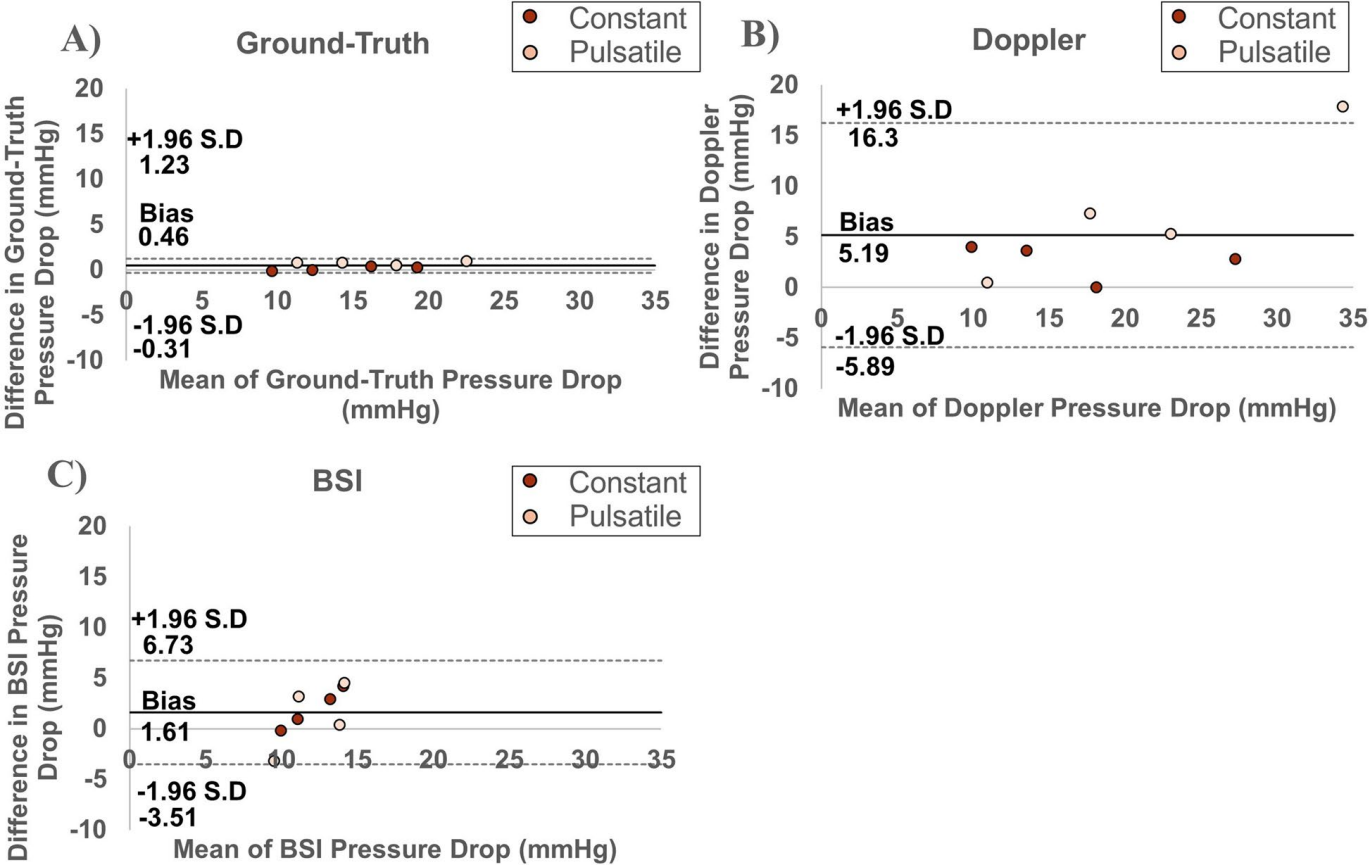
Bland–Altman plots illustrating the agreement between **A** ground-truth **B** Doppler and **C** Blood Speckle Imaging estimations of pressure drop across two days of experiments (*n* = 16). The solid line represents the bias within the methods, while the 95% limits of agreement (± 1.96 standard deviation) are represented by the dashed lines

Intra-technique reproducibility of the pressure drop estimations made across the two days of experiments by the three methods are presented in Fig. 9. Bland–Altman analysis produced statistically significant intra-technique bias values of 0.46 mmHg and 5.19 mmHg for ground-truth and Doppler, respectively (*p* < 0.05). The 1.61 mmHg bias for BSI was not statistically significant (*p* > 0.05). The upper and lower limits of agreement were 1.23 mmHg and -0.31 mmHg, 16.3 mmHg and -5.89 mmHg, 6.73 mmHg and -3.51 mmHg for ground-truth, Doppler and BSI, respectively (Fig. 9).

## Discussion

This study provides evidence to show that both BSI and Doppler techniques can make accurate estimations of low pressure drops in a controlled and reproducible aortic phantom. However, for stenotic conditions of clinical relevance in the setting of aortic stenosis, BSI underestimates while Doppler overestimates the pressure drop.

The assessment of the pressure drop by echocardiography in conventional clinical practice is subject to important methodological limitations that cannot be solved by current BSI technology. Significantly different pressure drop estimations, large percentage errors, bias values and wide limits of agreement exist for Doppler and BSI when compared with ground-truth pressure drop estimations. This is coupled with poor intra-technique reproducibility across two days of experiments. These findings illustrate that despite its theoretical advantages, further development of BSI or alternative novel and more comprehensive methods for pressure drop estimation are required to improve clinical practice.

### Pressure drop estimation

A good agreement between pressure sensors, Doppler and BSI was found at low stenosis levels (10.5 ± 1.00 mmHg) but BSI significantly underestimated pressure drop at the next stenotic condition tested (13.3 ± 1.20 mmHg; Fig. 6). With the onset of pressure drop underestimations using BSI occurring at these low stenotic conditions, BSI in its current form is inappropriate for the classification of aortic stenosis severity, which begins at 20 mmHg [16]. BSI would likely be accurate in the estimations of trans-mitral valve pressure drop, where the upper classification limit is 10 mmHg [17]. That said, when comparing Doppler and BSI to ground-truth pressure drop estimations, the limits of agreement are wide for both methods (upper limit 14.6 mmHg, lower limit -6.74 mmHg for Doppler vs. upper limit 2.82 mmHg, lower limit -9.43 mmHg for BSI; Fig. 8). Over/underestimations of transvalvular pressure drop by these margins are clinically significant as they could lead to incorrect diagnoses and the misclassification of disease severity. The differences between the experimental conditions should only be attributed to the ability to capture the peak velocity events, since both methods used the simplified Bernoulli formulation to estimate pressure drop. Pressure sensors were used to demonstrate that comparing ground-truth pressure values, acquired using pressure sensors, to velocity-based estimations of pressure drop results in discrepancies.

At the higher pressure drops, with higher flow velocities, BSI significantly underestimates pressure drop (Fig. 6). A negative linear relationship is observed for absolute error (Fig. 7) and agreement (Fig. 8B). Absolute error at the highest flow rate was significantly different to that at the lowest flow rate (Fig. 7). Underestimation of pressure drop with BSI is therefore more pronounced at higher flow rates. These results are consistent with previous findings conducted in vitro*/*in silico*,* whereby BSI was shown to underestimate flow velocity [18–21]. The largest in vivo study to date was performed in 51 healthy paediatric controls, where underestimations of velocity values acquired using BSI were also observed. The same study also revealed that the difference tended to increase at higher velocities [8]. High velocity gradients and considerable out-of-plane flow generated across flow obstructions lead to speckle decorrelation [18, 22]. This explains the underestimation of pressure drop at the higher flow rates using BSI.

On the other hand, pressure drops estimated using Doppler were significantly higher than ground-truth pressure drop at the greatest level of stenosis tested (20.9 ± 1.92 mmHg). This is likely due to the error in estimation of momentum from a single velocity value: the characterisation of the pressure drop requires the full velocity profile [3, 5]. This finding has clinical significance as a pressure drop of 20 mmHg is the lower limit for the classification of moderate aortic stenosis [16]. Significant overestimation of pressure drops in this range could result in misclassification of aortic stenosis severity and the inappropriate treatment of patients. The bias of Doppler measurements across the experimental conditions was 3.92 mmHg (*p* < 0.05); with peak overestimations of up to 20 mmHg (Fig. 8A). These findings are consistent with those reported by Donati et al. (2017), where pressure drop values obtained using the Simplified Bernoulli formulation were shown to overestimate the true pressure drop by 54% [3].

The peak pressure drop values measured by the pressure sensors in the phantom are different to net pressure drops measured in clinical practice during cardiac catheterisation, which measure the pressure difference between the left ventricular outflow tract and the ascending aorta, downstream of the vena contracta [23]. The peak pressure drop measured by the pressure sensors in the phantom is at the location of the vena contracta, as is the case for the Doppler data. The effect of pressure recovery further downstream, which is the traditional understanding of the reason of pressure drop overestimation by Doppler, can therefore be excluded.

**Fig. 10:**
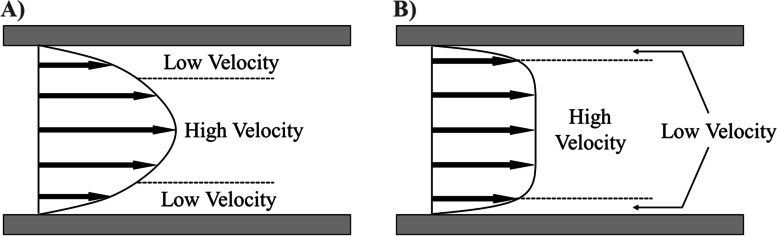
A diagram illustrating velocity profiles in regions of **A** high flow and **B** low flow

The absolute error of measurements made using Doppler increased with flow rate and a significant difference in absolute error was observed between the highest and lowest flow rates (Fig. 7). A linear increase in the difference between the estimated pressure drop of Doppler vs. ground-truth with increasing level of stenosis can also be observed in the respective Bland-Alman agreement plot in Fig. 8A. These findings support the observation that the overestimation of pressure drop by Doppler echocardiography is more pronounced at higher flow rates. The simplified Bernoulli formulation is accurate for uniform spatial velocity profiles (i.e. at the cross-section), observed at low flow velocities [3]. As flow rate increases, the spatial flow profiles, driven by viscous effects, become sharper and more paraboloidal in shape (Fig. 10A), deviating from the flatter spatial flow profiles, driven by inertial effects, observed at low flow rates (Fig. 10B). Donati et al*.* (2017) report that the variable deviations from the flat velocity profile cause an uncontrolled source of overestimation of pressure drop when applying the simplified Bernoulli equation (Supplemental material C-D of Donati et al*.* 2017) [3]. Our results report that the high velocity regimes, driven by viscous effects, gradually introduce a larger overestimation. This explains why Doppler is less accurate under these higher flow regimes (Fig. 7). Further variability would be expected if different valve models and geometries were studied, or indeed if using in vivo data. These conditions would likely increase the degree of mismatch between true and estimated pressure drops further.

As a final minor remark, no significant difference was observed between the mean pressure drops achieved under constant and pulsatile conditions, allowing them to be grouped within each pump flow rate (Fig. 6; *n* = 4).

### Intra-technique reproducibility

The intra-technique reproducibility analysis demonstrates a small, but statistically significant, bias of the ground-truth pressure readings (Bias = 0.46 mmHg (*p* < 0.05), upper limit 1.23 mmHg, lower limit -0.31 mmHg; Fig. 9A). Albeit small, this significant bias should be considered when interpreting the agreement between Doppler and BSI vs. pressure sensors. Variability is greater in the pulsatile estimations despite the fact each pressure drop is calculated as the mean over 6 cycles. BSI is less variable than Doppler (Bias = 1.61 (*p* > 0.05), upper limit 6.73 mmHg, lower limit -3.51 vs Bias = 5.19 mmHg (*p* < 0.05), upper limit 16.3 mmHg, lower limit -5.89, respectively; Fig. 9B-C). However, the reproducibility of BSI may be positively influenced by its inability to track high pressures, resulting in false clustering of measurements (Fig. 9C). The reproducibility of Doppler measurements is influenced by large disagreement at the greatest pulsatile flow rate (Fig. 9B). Disagreement also exists in the ground-truth measurements under this condition (Fig. 9A), which may account for intrinsic variability of the experimental conditions and not the measurement devices, and should be considered when interpreting the reproducibility of Doppler pressure drop measurements at higher flow rates.

### Limitations

These experiments were performed using a single model of a healthy aortic valve. The ultrasound probe was placed directly against the phantom to make the BSI and Doppler acquisitions with low penetration depth. These two factors represent a best-case scenario for the acquisition data for pressure drop estimation. In human subjects, acquisitions would be at an increased penetration depth, thus reducing the imaging frame rate, with an increased level of attenuation. In addition, experiments in human valves would exhibit more physiological and/or pathological variation. The results, therefore, would likely be different if data were obtained in vivo*.*

Although qualitative evidence (Fig. 5 and Additional File 1) and quantitative evidence (Fig. 6) demonstrate plausible haemodynamic behaviour, it is important to consider that similarity between the mechanical properties of the silicone valve and those of a human valve is not demonstrated beyond the reasonable confidence reported previously [12, 13].

In these experiments, the velocity profile and resultant pressure drop were controlled by modifying the pump flow rate. Changing the valve type or orifice area would be the ideal workbench for this experiment as it changes the velocity profile under the same flow conditions. However, this was not performed in order to avoid damaging the valves during the changeover procedure, and to maximise the reproducibility of the pressure drops created by a fixed valve. Additional experiments with different pulsatile conditions, using different frequencies and duty cycles, would allow for a better understanding of transient effects during the upstroke/downstroke of the acceleration of the jet, and thus secondary effects of the impact of the temporal resolution of data. The lack of these results is a limitation of the study.

In this work, pressure drop estimations were made using a single peak velocity value acquired by BSI. The reported limitations of current BSI technology preclude the use of pressure estimations made using the full velocity profile at this stage as underestimation will exist across the region of interest where higher flow velocities are found. Future technological advances and resultant improvements in temporal resolution may improve the ability of BSI to track higher flow velocities and therefore estimate greater pressure drops more accurately, allowing for two advantages, to correctly account for the physics of advection by capturing the full velocity profile [3], and to overcome the angle-dependence and aliasing limitations of Doppler echocardiography.

Given the small sample size, the results of this pilot study should be considered as preliminary. Future in vivo studies are required before BSI can be used in the clinical setting.

## Conclusions

BSI accurately estimated pressure drops up to 10.5 mmHg in controlled and reproducible phantom conditions of low stenotic burden, which may be useful for estimations of trans-mitral valve and intra-cardiac pressure drops. BSI underestimated all of the greater pressure drops tested, likely due to an inability of the algorithm to track higher flow velocities and speckle decorrelation. Doppler overestimated pressure drop values of clinical significance, in line with the published literature. Although BSI offers a number of theoretical advantages to conventional Doppler echocardiography, further refinements and clinical studies are required with BSI before it can be used to improve transvalvular pressure drop estimation in the clinical evaluation of aortic stenosis. 

## Supplementary Information

**Additional file 1.** Example BSI Acquisitions. A video file containing examples of the BSI acquisitions at each of the flow rates studied.

## Data Availability

The datasets generated and/or analysed during the current study are available in the figshare repository [10.6084/m9.figshare.20237469.v2].

## Abbreviations

BSI: Blood speckle imaging
CT scan: Computerised tomography scan
